# Moderate physical exercise and ATP modulate the P2X7 receptor and improve cisplatin-induced gastric emptying delay in rats

**DOI:** 10.1590/1414-431X2024e13234

**Published:** 2024-05-03

**Authors:** Y.A. Gomes, W.L.L. Santos, C.S. Pinheiro, J.S. Severo, J.C.C. Oliveira, A.C.A. da Silva, B.L.B. dos Santos, C.H.L. Rocha, A.A. dos Santos, M.T.B. da Silva

**Affiliations:** 1 Programa de Pós-Graduação em Farmacologia, Universidade Federal do Piauí, Teresina, PI, Brasil; 2 Programa de Pós-Graduação em Alimentos e Nutrição, Universidade Federal do Piauí, Teresina, PI, Brasil; 3 Laboratório de Exercício e Trato Gastrintestinal, Departamento de Educação Física, Universidade Federal do Piauí, Teresina, PI, Brasil; 4 Oncoclínica, Teresina, PI, Brasil; 5 Departamento de Fisiologia e Farmacologia, Faculdade de Medicina, Universidade Federal do Ceará, Fortaleza, CE, Brasil; 6 Departamento de Imuno-Fisiologia e Farmacologia, Laboratório de Fisiologia, Centro de Investigação Farmacológica e Inovação Medicamentosa, (MedInUP), Instituto de Ciências Biomédicas Abel Salazar (ICBAS), Universidade do Porto, Porto, Portugal

**Keywords:** ATP, Brilliant Blue G, Cisplatin, Physical exercise, Gastric emptying

## Abstract

Patients undergoing chemotherapy with cisplatin commonly present gastrointestinal effects such as constipation and gastric emptying (GE) delay. Both the purinergic system and physical exercise modulate the gastrointestinal (GI) tract. In the current study, we investigated the role of ATP, physical exercise, and P2X7 receptor blocking on GE delay induced by cisplatin in rats. Male rats were divided into the following groups: control (C), cisplatin (Cis), exercise (Ex), Brilliant Blue G (BBG), ATP, Cis+Ex, Cis+ATP, Cis+BBG, Cis+Ex+BBG, Cis+Ex+BBG+ATP, and Cis+ATP+BBG. GE delay was induced by treatment with 1 mg/kg cisplatin (1 time/week for 5 weeks, *ip*). The moderate physical exercise was swimming (1 h/day, 5 days/week for 5 weeks). At the end of the treatment or exercise and 30 min before the GE assessment, some groups received BBG (50 mg/kg, *sc*) or ATP (2 mg/kg, *sc*). Then, GE was assessed after a 10-min postprandial period. Chronic use of Cis decreased GE delay (P<0.05) compared to the control group. Both exercise and ATP prevented (P<0.05) GE delay compared to Cis. The pretreatment with BBG significantly inhibited (P<0.05) the effect of exercise and ATP. On the other hand, the association between exercise and ATP reversed (P<0.05) the effect of the BBG and prevented GE delay. Therefore, we suggest that both exercise and treatment with ATP activate P2X7 receptors and prevent GE delay induced by cisplatin in rats.

## Introduction

Chemotherapy with cisplatin is associated with gastrointestinal (GI) toxicity that can lead to many disorders such as reduction in body weight, alimentary problems, abdominal pain, intestinal inflammation, and constipation, among others. GI disorders caused by cisplatin stem from its ability to delay gastric emptying (GE). This delay often leads to post-meal abdominal discomfort, ultimately inducing nausea and vomiting (1). In light of this, various pharmacological and non-pharmacological approaches have been developed to enhance the quality of life of patients on cisplatin therapy.

One of the explanations for cisplatin-induced GI symptoms is mediation by the purinergic system (2). The purinergic system is made up of a large family of receptors, including P2X7, an ion channel modulated via adenosine triphosphate (ATP), which can be located in many types of cells and tissues such as adipocytes, macrophages, and bone tissue. The P2X7 receptor is associated with inflammasomes, which, after being activated via ATP, initiate an inflammatory cascade linked to the release of cytokines such as interleukin 1 (IL-1) and IL-18, affecting GI permeability and motility (3). The inhibition of the P2X7 receptor may be associated with some pathophysiological disorders in many systems such as endocrine, cardiovascular, and GI. Brilliant Blue G (BBG) is an antagonist of the P2X7 receptor that can be used as a therapeutic approach in neurological and inflammatory diseases (4).

The effectiveness of pharmacological and non-pharmacological therapies in treating or preventing cisplatin-induced GI toxicity is also important. Physical exercise is a non-pharmacological therapy that can be used to prevent collateral effects during chemotherapy treatment. In gastrointestinal cancer patients undergoing chemotherapy, physical exercise decreases the incidence of nausea and acid reflux and alleviates fatigue and appetite loss (5). Moreover, physical exercise is an effective strategy to mitigate the side effects associated with chemotherapy by reducing intestinal inflammation, restoring the integrity of the intestinal barrier, and regulating the microbiota (1,5,6). Several mechanisms are involved in the attenuation of cisplatin-induced GI symptoms by exercise, such as regulation of GI motility and modulation of the neuro-endocrine axis (6). However, little is known about the effects of physical exercise and modulation of the purinergic system and their association with cisplatin-induced GI dysmotility. We hypothesized that exercise and ATP can activate the purinergic system via the P2X7 receptor and modulate gastric dysmotility induced by cisplatin in rats.

## Material and Methods

### Animals and ethical approval

**Figure 1:**
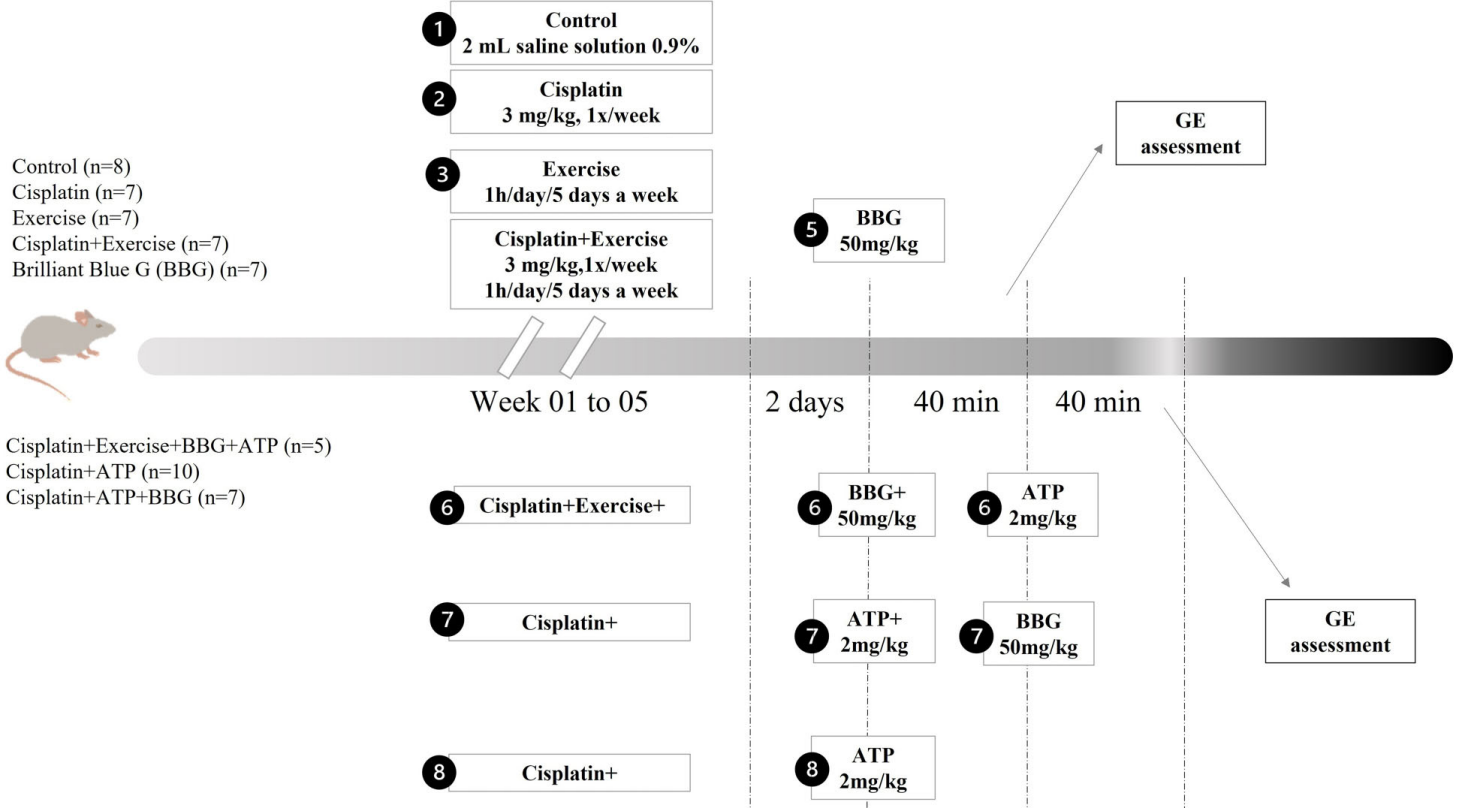
Experimental design. ATP: adenosine triphosphate; BBG: Brilliant Blue G; GE: gastric emptying.

Male Wistar rats weighing between 230-250 g were obtained from the Federal University of Piauí, Brazil. The animals were housed in collective cages with water and feed *ad libitum* with controlled temperature (28±2°C) and a 12-h light/dark cycle. All procedures were performed according to the recommendations of the “Guide for the Care and Use of Laboratory Animals” and were approved by the Ethics Committee on Animal Use (CEUA) of the Federal University of Piauí (Protocol 431/18). Rats were separated into control (n=8), cisplatin (n=7), exercise (n=7), cisplatin+exercise (n=7), Brilliant Blue G (BBG) (n=7), cisplatin+exercise+BBG,+ATP (n=5), cisplatin+ ATP (n=10), and cisplatin+ATP+BBG (n=7) groups. Figure 1 presents the experimental design of the study.

### Induction of gastrointestinal disorders

GE delay was induced according to Silva et al. (1). We used cisplatin (Citoplax^®^ 50 mg/50 mL, Bergamo Ltda., Brazil). The induction protocol consisted of cisplatin administration (3 mg/kg, *ip*) once per week for 5 weeks. The control rats received only 0.9% saline solution via *ip*. All groups treated with cisplatin also received 2 mL of 0.9% saline to prevent chemotherapy-induced nephrotoxicity.

### Physical exercise protocol and pharmacological treatment

We used a moderate-intensity exercise protocol (swimming) as described by Silva et al. (1). Initially, all rats underwent a period of adaptation to water before training. Physical exercise and/or cisplatin treatment were started simultaneously. The exercise was performed in collective tanks (100 cm long × 80 cm wide × 80 cm deep) with a maximum of 4 rats and water at a depth of 50 cm, and maintained at a controlled temperature of approximately 30±2°C. The protocol consisted of swimming with a load of 5% body weight attached to the tail (1 h/day, 5 days/week for 5 weeks). The sedentary rats were subjected to contact with shallow water without physical exercise to account for any stress bias caused by being in contact with water. On the experimental day, 40 min before GE assessment, a separate subset of rats in the Control and Exercise groups received ATP (2 mg/kg *ip*) and/or BBG (50 mg/kg, *sc*) according to de Oliveira et al. (4).

### Assessment of gastric emptying

After the last session of exercise and/or treatment, the rats were subjected to 18 hours of fasting. After 24 h, the rats were gavage-fed with a liquid test meal that consisted of 1.5 mL of 50 mg/mL phenol red in a 5% glucose solution. After a 10-min postprandial interval, the rats of all groups were sacrificed by thiopental overdose (100 mg/kg, *ip*). GE was assessed according to Lima et al. (7).

### Statistical analysis

The Shapiro-Wilk test was employed to assess data normality, and the results of each group are reported as means±SE. For comparison between groups, we performed a one-way analysis of variance (ANOVA) followed by the Tukey test. The difference was considered significant if the P-value was <0.05 (95% confidence interval).

## Results

**Figure 2:**
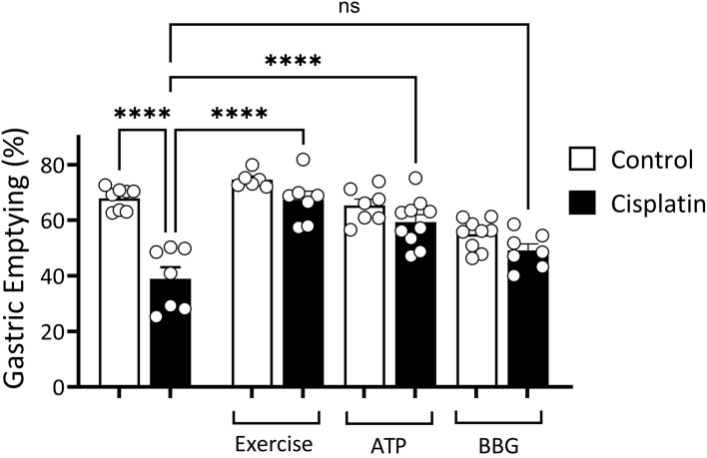
Effect of physical exercise, adenosine triphosphate (ATP), or Brilliant Blue G (BBG) on gastric emptying delay induced by cisplatin in rats. The rats were gavage-fed (1.5 mL) the test meal (phenol red in glucose solution) and euthanized 10 min later to determine gastric dye recovery by spectrophotometry. Data are reported as means±SE. ****P<0.05, one-way ANOVA with Tukey *post hoc* comparisons. ns: not significant. Control rats received 0.9% saline solution, *ip;* cisplatin rats received 1 mg/kg cisplatin (1 time/week for 5 weeks, *ip*).

Figure 2 shows the results of the effect of exercise, ATP, and BBG on GE delay induced by cisplatin. We observed a significant decrease in GE in the cisplatin rats compared with the control rats (38.8±4.1 *vs* 67.8±1.4%). On the other hand, a significant decrease (P<0.05) was found in exercise+cisplatin and ATP+cisplatin groups compared with the cisplatin rats (67.3±3.1 and 59.3±2.6 *vs* 38.8±4.1%). Moreover, we did not observe differences between BBG+cisplatin compared with cisplatin rats (49.1±2.4 *vs* 38.8±4.1%). BBG alone significantly decreased GE (P<0.05) compared with the control rats (54.9±1.8 *vs* 67.8±1.4%).

**Figure 3:**
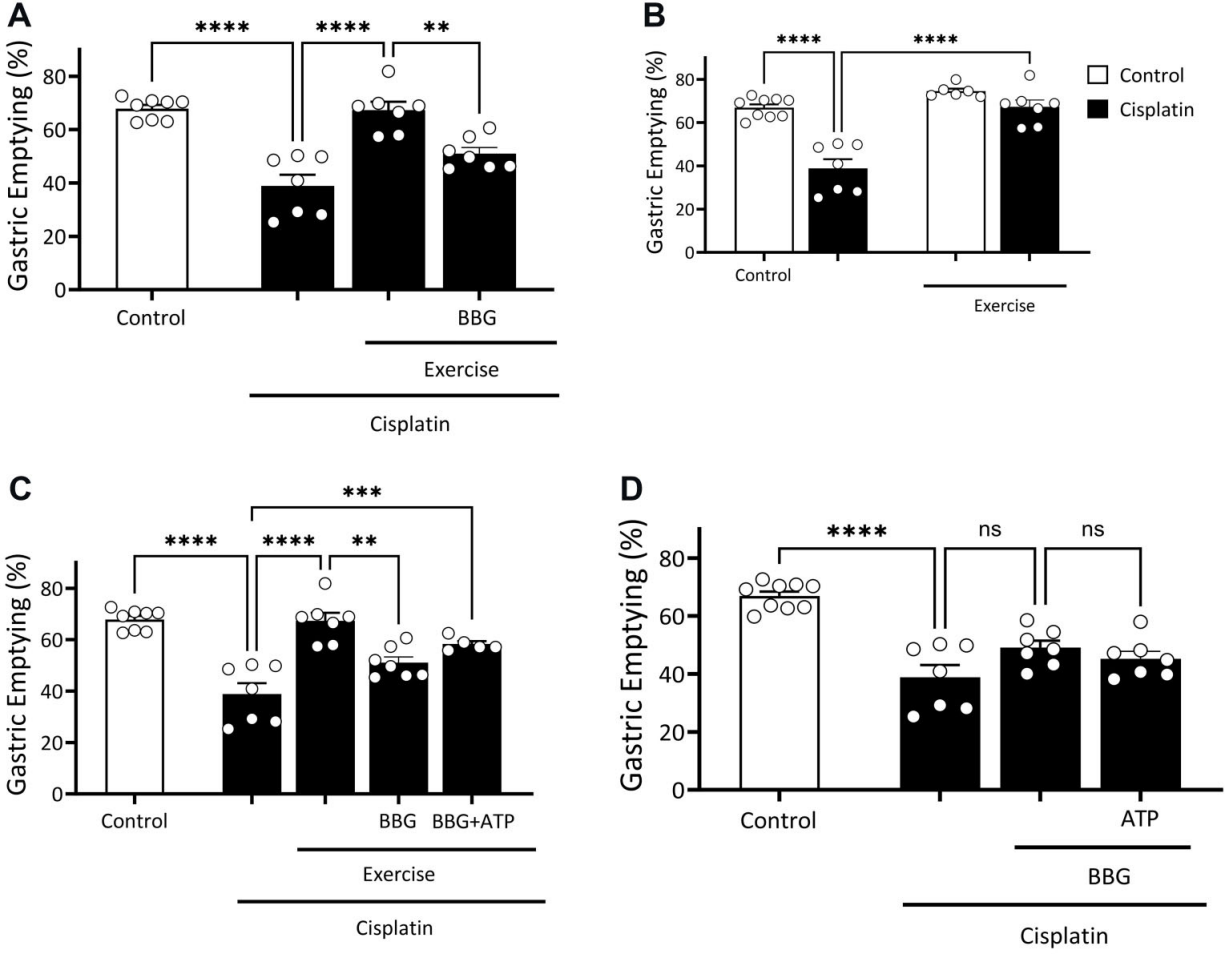
Effect of combined physical exercise, adenosine triphosphate (ATP), or Brilliant Blue G (BBG) on the gastric emptying delay induced by cisplatin in rats. **A**, Exercise+BBG groups, **B**, Cisplatin+Exercise groups, **C**, Exercise+BBG+ATP groups, and **D**, BBG+ATP groups. The rats were gavage-fed (1.5 mL) the test meal (phenol red in glucose solution) and euthanized 10 min later to determine gastric dye recovery by spectrophotometry. Data are reported as means±SEM. **P<0.01, ***P<0.001, ****P<0.0001, one-way ANOVA with Tukey *post hoc* comparisons. ns: not significant.

Figure 3A depicts the effect of cisplatin, cisplatin+exercise, and cisplatin+exercise+BBG on GE delay. In cisplatin+exercise+BBG rats, we observed a decrease in gastric emptying (P<0.05) compared with the cisplatin+exercise rats (51.0±2.2 *vs* 67.3±4.1%). We did not observe differences between the cisplatin and cisplatin+exercise+BBG groups. Figure 3B shows a similar preventive effect by reducing GE (P<0.05) in the cisplatin+exercise group compared to the cisplatin group (67.3±3.1 *vs* 38.8±4.1%). Figure 3C shows a preventive effect (P<0.05) of GE delay in the cisplatin+exercise+BBG+ATP group compared to the cisplatin group (38.8±4.1 *vs* 58.2±1.1%). Figure 3D shows that cisplatin+BBG and cisplatin+BBG+ATP did not prevent delays induced by cisplatin.

## Discussion

In this study, we observed that GE delay induced by cisplatin can be regulated by exercise and ATP administration. We speculated that both ATP and exercise modulated the purinergic system by the P2X7 receptor. We initially hypothesized that the P2X7 receptor also had a role in mediating cisplatin-induced GE delay. To investigate this relationship, we administered ATP, an endogenous ligand that has high affinity with the P2X7 purinergic receptor, and/or BBG, a selective P2X7 receptor antagonist (8).

We observed cisplatin-induced dysautonomia, characterized by increased sympathetic tone and reduced vagal tone, a phenomenon that is related to GI disorders. Furthermore, cisplatin can induce changes in serotonin secretion by enterochromaffin cells, which can be active 5-HT_3_ and 5-HT_4_ receptors, inducing relaxation of gastric muscles and gastric dysmotility (1).

The P2X7 receptor seems to be involved in GE control, as previously demonstrated by de Oliveira et al. (4), and is expressed in the GI musculature of rats (9,10). The mechanisms are still not clear, but likely involve inflammation, redox signaling, and release of serotonin (4).

One agonist of the P2X7 receptor, ATP, has also been recognized as a target of chemotherapy. Several chemotherapeutics, such as cisplatin, act on the ATP release of tumor cells, depleting the content of intracellular ATP, which favors apoptosis (11,12). Cisplatin is a first-line drug in the treatment of gastric cancer. Concomitantly, P2X7 expression and the derived cytokine IL-18 have been recognized as gastric cancer biomarkers (13,14). Thus, the results suggested that ATP administration may be involved in the modulatory activity of P2X7, which can reverse the GE delay induced by cisplatin.

Moreover, Li et al. (15) suggested that cisplatin treatment reduces acetylcholine (ACh) concentration and decreases the expression of its receptor, as well as the ACh activity in the gastric tissue, inducing damage to the Interstitial cells of Cajal (ICCs) and affecting GE. Thus, ICCs play a fundamental role in GI function associated with neurotransmitters involved in controlling GI contractility (16). As ATP may act as a neurotransmitter for inhibitory enteric neurons, which stimulate ICCs (17), this could explain the reversal of GE found in this research.

BBG is a P2X7 receptor antagonist (4). We observed that P2X7 alone inhibited GE. Moreover, in rats with gastric dysmotility induced by cisplatin, the effect of the GE delay remained. Therefore, we suggest that chronic treatment with cisplatin may induce purinergic signaling via the P2X7 receptor, similar to the inhibition with BBG.

Physical exercise was able to reverse the GE delay induced by cisplatin. Miron et al. (18) observed that the practice of exercise protocols positively regulated the activity of purinergic enzymes and purinoceptors, notably P2X7R and that exercise blocked the modulation in the signaling proteins of the P2X7 receptor cascade in many structures in the brain, inducing neuroprotective effects and anti-inflammatory actions. In inflammatory diseases, the neuroprotective effect may be related to adenosine receptors, such as A2AR, which confers neuroprotection in neurodegenerative diseases.

Furthermore, physical exercise may restore the normal autonomic balance altered by cisplatin. This dysautonomia induced by cisplatin may be associated with sympathetic hyperactivity and a decrease in vagal tone, which induces repercussions in the GI tract, in particular GI motility (19). In this sense, we suggest that exercise has the potential to augment the availability of ATP (20), which can, in turn, exert indirect effects on the aforementioned aspects modulating GE.

When we analyzed the co-intervention with BBG and exercise or ATP administration, each intervention prevented GE delay induced by cisplatin. Thus, we suggest that ATP-activated P2X7 prevented GE delay, and physical exercise might indirectly increase ATP circulation and activate these receptors, improving gastric dysmotility.

In conclusion, the results reported indicated that chronic treatment with cisplatin inhibited the P2X7 receptor and induced GE delay. ATP treatment directly activated the P2X7 receptor and prevented this delay, while exercise released even more ATP-improving GE delay induced by cisplatin.
